# A multi-centre qualitative study exploring the experiences of UK South Asian and White Diabetic Patients referred for renal care

**DOI:** 10.1186/1471-2369-13-157

**Published:** 2012-11-23

**Authors:** Emma Wilkinson, Gurch Randhawa, John Feehally, Ken Farrington, Roger Greenwood, Peter Choi, Liz Lightstone

**Affiliations:** 1 Institute for Health Research, University of Bedfordshire, Luton, UK; 2 John Walls Renal Unit, Leicester General Hospital, Leicester, UK; 3 Lister Renal Unit, North Herts NHS Trust, Stevenage, UK; 4 West London Renal and Transplant Centre, Imperial College Healthcare NHS Trust, London, UK

**Keywords:** South Asian, Patient experience, Renal complications of diabetes, Access, Care pathway

## Abstract

**Background:**

An exploration of renal complications of diabetes from the patient perspective is important for developing quality care through the diabetic renal disease care pathway.

**Methods:**

Newly referred South Asian and White diabetic renal patients over 16 years were recruited from nephrology outpatient clinics in three UK centres - Luton, West London and Leicester – and their experiences of the diabetes and renal care recorded.

A semi-structured qualitative interview was conducted with 48 patients. Interview transcripts were analysed thematically and comparisons made between the White and South Asian groups.

**Results:**

23 South Asian patients and 25 White patients were interviewed. Patient experience of diabetes ranged from a few months to 35 years with a mean time since diagnosis of 12.1 years and 17.1 years for the South Asian and White patients respectively. Confusion emerged as a response to referral shared by both groups. This sense of confusion was associated with reported lack of information at the time of referral, but also before referral. Language barriers exacerbated confusion for South Asian patients.

**Conclusions:**

The diabetic renal patients who have been referred for specialist renal care and found the referral process confusing have poor of awareness of kidney complications of diabetes. Healthcare providers should be more aware of the ongoing information needs of long term diabetics as well as the context of any information exchange including language barriers.

## Background

Previous studies in the UK have identified a greater relative risk for type 2 diabetes related end-stage renal disease (ESRD) in South Asians (those originating from India, Pakistan, Bangladesh, and Sri Lanka) [1,2], and preliminary evidence has suggested that quality of health care for South Asians is inadequate and compliance poor [3,4]. There is also a low-uptake of hospital-based diabetes services, with growing evidence that South Asians are subsequently referred later for renal care, and are more likely to be lost to follow-up [5]. Moreover, there is evidence that knowledge of diabetes and its complications is poor among South Asians [4,6].

National Service Frameworks for Diabetes and Renal Services were introduced in the UK in 2002 and 2006 respectively. These Frameworks provide guidance to commissioners and providers of health care commissioners about the minimum standards of care that should be offered across the UK. Significantly, the Frameworks recognised the disparity between ethnic groups and promoted a focus on earlier detection and ethnicity as a risk factor to improve outcomes for diabetic renal disease across different population groups [7,8]. Furthermore, the introduction of the Quality Outcomes Framework indicators in primary care for diabetes in 2004 and estimated glomerular filtration rate (eGFR) reporting in 2007 were both infrastructure developments introduced to improve for quality of care for all diabetes patients [9-11].

The research described here was one element of a larger study, the diabetic renal disease care pathway study, which explored the concept of patient access to quality care i.e. how patients gain access to diabetes and renal services and how services are perceived by patients. The premise being that services need to be relevant and effective if the population is to have access to improved health outcomes. By combining audit and interview methods the larger project aimed to investigate whether there were differences between the South Asian and White patient populations in referral rates, indicators for type 2 diabetic renal disease, medication management, as well as patient attitudes to, and experience of, services at key points through the care pathway.

This paper describes the qualitative analysis of patient interviews in which White and South Asian type 2 diabetic renal patients who had been recently referred to renal services talked about their experiences of diabetes and renal care. The results identify themes that relate to access on an individual level – reaction to referral; understanding at referral; understanding prior to referral; influence of comorbidities and influence of ethnicity – which provide a picture of patient experience of the diabetic renal disease care pathway at referral for these two groups. The analysis identified some aspects of care where differences between the two groups are linked to ethnicity as well as others where a more universal cultural dimension is operating.

## Methods

### Patient sample and recruitment

This qualitative element of the larger study was designed to capture the lived experience of patients who made up the sample of an audit of new referrals to renal care at three sites in 2007 and which is reported elsewhere. Semi structured individual interviews were the preferred data collection method because they enabled each patient’s experience to be captured individually.

All patients of South Asian or White European origin who were over 16 years old with a diagnosis of type 2 diabetes prior to referral and accepted for clinical review at specialist nephrology departments in Luton, Leicester and West London during 2007, were identified from computerised patient information systems. Patients were approached by their nephrologist if they fulfilled the inclusion criteria, had had at least one specialist renal consultation following referral, and if the nephrologist considered them in good enough physical and mental health to take part.

It was estimated that up to 20 patients (10 White and 10 South Asian) would be recruited at each site (up to 60 in total), and that this would collectively provide: a representative sample of patients being referred to specialist renal services; coverage of the predominant groups of South Asians in the UK: Indian Gujarati; Indian Punjabi; Pakistani, and Bangladeshi; and an adequate sample for the proposed analysis. The latter being determined through experience, from within the team and elsewhere, of exploratory qualitative research and nonprobablistic sampling taking into account the overall study design, data collection method, and resources [12,13]. Moreover, as an objective was to understand patient experience of renal services, only patients who had attended at least one consultation with the renal team were included in the interview sample.

The project was approved by the NHS Local Research Ethics Committee. Recruitment took place in 2008 at the outpatient clinic or via postal correspondence if this was not possible. A member of the research team, which included bilingual researchers, was on hand at clinics to respond to any immediate patient queries, and patients were able to speak to a member of the research team by telephone prior to taking part if they required further information. All patients approached received detailed verbal and written information about the aims and objectives of the research. Recruitment method and documents: invitation letter to patient; patient information sheet and consent form were approved as part of the ethics review.

### Interview format

A semi-structured questionnaire schedule was developed specifically for the purpose of this study. This was devised by collaborating researchers (social scientists and clinicians) and comprised a series of questions with prompts covering the following broad areas: diagnosis of renal complications; access to and experience of renal services; current health; disease management; impact and support; access to information and communication. Both the patient information sheet and the preamble to the interview asked patients to recount their experience, in their own words, and the interview schedule was intended to be used as a guide and to ensure the main areas were covered during the course of the dialogue.

One to one interviews were conducted by researchers in the patient’s preferred venue, invariably the patient’s home, and in the patient’s language of choice, employing bilingual researchers where this was required. Interviews lasted between 40 minutes to one hour and were tape recorded. The resulting recordings were transcribed verbatim into Word documents.

### Analysis

Interview transcripts were repeatedly read through, an initial framework of key themes (initial thematic categories) was formulated and interviews were analysed using these themes as well as others as they emerged. Although broad interview areas had been determined a priori these themes were identified retrospectively through the analysis process. Thematic approach to analysis is a widely used process in the analysis of qualitative data [14,15] and was used in this research to identify a framework of themes and sub themes which relate to the access and quality of healthcare. Nvivo 7, a computer software package for qualitative research was used to facilitate data coding and retrieval. The lead researcher (EW) conducted the coding and data analysis in collaboration with bilingual research interviewers who provided feedback on individual interviews, and the chief investigator (GR) who had oversight of the process. Analysis of data from some of these themes (paragraph headings) forms the basis of the following results and discussion.

### Ethical approval

This study was given ethical approval by Bedfordshire NHS Research Ethics Committee in June 2005 - **REC reference number**: **05**/**Q0202**/**24**.

## Results

### Patient description

48 patients in total were recruited and interviewed, comprising 23 South Asian patients (14 men and 9 women) and 25 White patients (16 men and 9 women). The mean age of the patients was 70.3 years (range 34–86 years) with a mean age of 67.4 years (range 34–79 years) for the South Asian patients and 72.8 years (range 51–86 years) for the White patients which was non significant (sig 0.056).

A third of the interviews were conducted by bilingual interviewers and were conducted either fully or in part in the interviewees preferred South Asian language, the rest of the interviews were conducted in English by the lead researcher.

Patients’ experience of diabetes ranged from a few months to 35 years with an average time since diabetes diagnosis of 14.7 years. Time since diabetes diagnosis was 12.1 years (range 6 months–35 years) and 17.1 years (range 2–40 years) for the South Asian and White groups respectively, which was a non significant difference (sig 0.076). A detailed descriptive account of the patient cohort from which this sample of patients was drawn can be found in a separate paper by these authors [16].

### Referral routes

Patients were referred to specialist renal services following investigation in another specialty or following management and monitoring in primary care.

### Reaction to referral

In recounting their experiences of being referred to renal services, patients articulated a variety of reactions: worry, fear, regret, ambivalence and disinterest. However, the most common response across both groups was confusion and this seemed to be associated in most instances with a lack of understanding of the problem being investigated.

‘*I*’*d like to know*, *more specifically*, *what the renal unit function is*. *I mean*, *I hated the word and yet it*’*s the word that*’*s referred to*, “*disease*”….*I think that the routine ought to be spelt out more definite*, *more positive*……*I don*’*t know what they*’*re looking at*, *I don*’*t know what they*’*re reading*, *presumably they might tell me*.’ WM1 72yrs

‘*We will go*… *my husband usually comes with me*. *He too doesn*’*t know much* (*English*). *When I go there*, *they will test my body*, *test my blood*, *my pressure*. *With that*, *it is over and then we go home*. *It*’*s been like this for the past one year*. *So I didn*’*t know what is happening or how is my body*. *It was the same with the GP too*.’ SAF2 61yrs

### Understanding at referral

Where patients had been referred as a result of an identifiable incident the referral reason was clear. However for the many other patients interviewed confusion about the reason for referral was associated with a lack of information:

‘*I know basically the function of the kidneys but renal problems*, *I don*’*t understand*. *And nobody seems to have the time to tell you*. *It would help*, *I think*, *if somebody*, *if the doctor himself can*’*t do it*, *that there*’*s somebody there in the health service than can explain why you*’*re being treated for renal problems*.’ WM3 72yrs

‘…*and then they say that* “*now you will be alright*”. *But nobody has told how much is working*…… *Not working properly*, *that*’*s alright*, *but nothing been said that how much it is working and how much it is not working*…. *The first time*. *He explained to me* – “*do you know how much it*’*s working*”? *I say*, “*I don*’*t know*, *nobody has told me*.‘ SAF1 69yrs

### Understanding prior to referral

For many patients referral to specialist services was the first time that renal complications of diabetes had been raised in their consciousness:

‘*To be honest with you I really didn*’*t know till I went to my last appointment with the doctor*, *Dr xxxxxxx*, *that was about a month ago*.’ SAM4 74yrs

‘*No*, *this is something recent*, *very recent*, *in the last 12 months*, *and I really wasn*’*t aware of it* …. *No*, *never thought about it*….*Yes*, *the eyes and the feet*, *because people talked about it quite a lot*.’ WF4 83yrs

‘*I think I would have been a different person if I*’*d known that from the beginning* … *because that really would have scared me*, *like the thought of something going wrong with my kidneys*, *like something going wrong with your lungs or your heart*, *it*’*s scary*. *So I think I would have* - *I probably wouldn*’*t even be on insulin now*.’ WF5 63yrs

This almost universal lack of awareness goes some way to explain the feelings of confusion described above as well as, for some, regretful reflection that had they been more aware of the potential risk of renal complications they may have been more careful with their diabetes management.

The absence of tangible symptoms compounded patients’ sense of bewilderment for those who were referred as a result of monitoring in primary care or an opportunistic test elsewhere.

‘*Well I did not really have any symptoms*, *I felt fine*. *I only found out that I had problems because I changed my G*.*P*, *if I had not changed G*.*P*: *then I would be none the wiser*.’ WM4 71yrs

‘*It doesn*’*t have any effect on me*, *no*…*you don*’*t know until it*’*s got a bit worse*….*or somebody else tells you how it*’*s progressing*, *yes*.’. WM5 70yrs

However it was notable that more South Asian patients reported symptoms and pain at referral to renal services compared to White patients:

‘*I am in pain but I don*’*t know if it*’*s my renal condition which is causing it*. *I have bad back pains*, *my stomach aches and my feet are very swollen*, *all this cause me difficulties*.’ SAM3 64yrs

‘*That*’*s when*, *my GP told me*, *because you have stomach pain continuously and you cannot climb up stairs*, *it may have been affected*…*kidney*…’ SAF2 61yrs

‘*Like I have now*, *in my knees*, *in my feet*. *My fingers feel numb*. *It is like the pain travels around my body*.’ SAF3 71yrs

### Influence of comorbidities

The majority of patients had comorbidities which were mainly cardiovascular, with a small number reporting cancer, COPD or arthritis (this is detailed in a separate paper) [16]. Comorbidities appeared to exacerbate the confusion at renal referral by making the scenario more complex, with multiple clinicians, medical prioritisation and bilateral communication between different specialties and the patient.

‘*Well yes*, *but the point is you forget half the time what the doctor*’*s for*. *I mean*, *like I*’*ve got Dr xxxxxx for the heart complaint and we used to have this young doctor*, *didn*’*t we*? *Then you*’*ve got Dr xxxxx for the other bit*, *then doctor what*’*s*-*his*-*name for the other bit and you think to yourself*, “*I*’*m seeing this one*, *what does he do*”, *you know*?’ WF2 76yrs

‘*But*, *again*, *I won*’*t get any proper advice from anybody*, *because when I was in the general hospital there was all practice doctors*, “*you shouldn*’*t do this*; *you shouldn*’*t do that*”. *But anyway I never argue with them*. *But again when I got back out*, *one says* “*do this*”, *another one says*, “*don*’*t do that*’; *so there you are*.’ SAM2 72yrs

In the context of renal referral, experience of cardiovascular and diabetic comorbidities did not contribute to a better understanding of the referral. The exception to this were a few patients who had been under the recent care of a diabetes specialist seemed to benefit from a better understanding of the monitoring process and from good communication with the consultant.

‘*The tests showed one of my kidneys is shrinking and working at 60*% *and the other kidney is working at 30*%. *I have been advised to take care*, *take my medication properly and to have a good diet with no sugar*. *I do all of this*, *what else can I do*? *If you advise me I will do it*.’ SAM5 64yrs

‘*Well from what I*’*ve gleaned at the clinic*, *as you get older your kidneys start to reduce anyway and with the diabetes and* – *it accelerates all that*…: *It*’*s not until you see someone like Dr xxxxxx that you really get the nitty gritty*…. *She doesn*’*t hold nothing back*; *she tells you exactly where you are*; *no brushing things under the carpet with her it*’*s just straight to the point*…. *Yeah*; *she*’*ll answer anything you ask*, *even if it*’*s not connected with diabetes but you might think it is*, *she will answer you*…’ WM6 70yrs

### Influence of ethnicity

The analysis described here was comparative, identifying themes across the two patient groups distinguished by their ethnicity. There was an underlying differential of 5 years in both age at referral and years with diabetes between the two groups which was non-significant.

Apart from this, results from this analysis show that the key dimension in which ethnicity has influence is in direct communication between patient and health care providers. Non-English speakers in our sample reported how they rely mainly on available family members to help communicate with service providers and that this is generally satisfactory for their needs for outpatient appointments. However there were additional problems which were encountered for some South Asian patients if they needed more involved or inpatient treatment. The following contrasts experience of the same treatment between a White and South Asian patient where language barrier affects access:

‘*I had iron infusions you know like injections*. *I had that three times over a week on the dialysis ward*, *I was an out patient but that was an eye opener as I saw many young people receiving dialysis and that gave me an insight and incentive to look after myself more*. *After the infusions I was okay*, *they seemed to correct it*……*I think they are doing a good job*. *I was impressed with the renal and dialysis unit*. *Like I said it was a wake up call when I have my iron Infusion injections as it was on the dialysis ward*. *I went there three times and saw the same people there*, *some were very young and that was frightening*.’ WF3 76yrs

‘*Like about my kidney*, *I should know*. *When I went to have the irons injected*, *I was really tensed dear*…*why did they inject this*, *why is the ambulance coming*…*you just think and see*…*I am fine and why is the ambulance coming to pick me up*, *why do they take me*, *why do they bring me back*, *who to ask*…*for the past one month I*’*ve been tensed* (*tension* - *worried*) *about this*. *That*’*s why I asked that girl* (*interpreter*), *why they injected this*…*and she said she wasn*’*t connected to this*, ‘*you don*’*t have irons*, *that*’*s why they injected this*’. *So I asked this doctor*, *why they injected this and she told me you don*’*t have red blood in your body*…*because it is not clean they had to inject this and other thing is* ‘*you have sugar for a long time right*, *because the sugar level became higher*, *you take medicines and this and that right*, *because of all that your kidneys have been affected*…’ SAF2 61yrs

## Discussion

Thematic analysis of patient interviews identified a small number of themes which described patient’s reactions to and associated experiences of referral to renal services. The comparison of the two ethnic groups across these themes indicated that there were more similarities than differences except where there were language barriers.

Referral of patients to specialist kidney services was either via another specialty or following management and monitoring in primary care. These are the routes which can impact on patient access and experience through the care pathway for diabetic renal disease [8].

Referral to specialist renal services was confusing for some patients. These patients reported limited understanding of the reason for referral, not having adequate information about their renal condition nor renal services when they were referred. This situation was compounded for patients with comorbidities and those who did not speak English.

Despite having familiarity and first-hand experience of living with diabetes patients had low awareness of the renal complications of diabetes. This suggests that there is a need for ongoing patient education for diabetics particularly with respect to renal complications as patients were less aware of these than of retinopathy and neuropathy.

The time since diabetes diagnosis was on average 14.7 years which is an extended timeframe over which patient awareness and ongoing education could take place. Where there was understanding of the monitoring process and access to clinicians willing to provide information, referral to kidney services was not so confusing but seen as an extension of this care. However few patients had this experience.

For South Asian patients the earlier onset of type 2 diabetes [17] and the shorter time before referral to renal care [16] suggests an even greater importance of this period for preventing the onset of renal complications for this group. It may be that diabetic kidney disease progresses more quickly in South Asians because of barriers to access and that risk is less well managed [18]. The observation that pain was reported by more South Asian patients than White patients also hints that there may be other factors associated with ethnicity which could affect the experience of symptoms and thereby the timing of access to health care. However this study is unable to explain this observation further.

The main difference in access between the two patient groups at referral appeared to be related to communication barriers.

Comparing and contrasting two broad groups whose longer term outcomes for ESRD show marked differences but whose lived experiences are product of context, past and present, and individual propensity to diabetes and renal complications, does as much to show inter-group similarities and intra-group heterogeneity, as it does to identify cross-group differences.

Here we have seen that within both groups there were patients whose experience of referral was associated with confusion, lack of understanding and information about renal complications of diabetes. We suggest that these relate to universal cultural issues concerning the communication of diagnoses and health related information in the context of renal services. The situation described was exacerbated for South Asian patients who were non-English speakers as there were additional communication barriers.

The association between culture and access is complex and conceptual. It is difficult to know when a person’s ethnicity makes a difference and mediates a person’s relationship with healthcare services as there are many other factors such as socioeconomic status, age, education which come into play [19]. Larger samples and collection of more detailed sociodemographic data would allow a more sensitive analysis by ethnicity and therefore better understanding of the influence of ethnicity and culture on access to diabetes and renal care.

### Limitations

The patient sample came from the sample for an audit of all diabetic patients who had been referred for renal care in the period January 2007 to January 2008 and who had attended at least one appointment with a renal specialist during that time. We did not recruit non-attenders or those who may have not been given an appointment but returned to primary care with advice from renal specialist to general practitioner.

The sample size estimate was made before the study started to enable the planning process and was based on renal consultants experience their patient population of realistic recruitment numbers. The sample frame was all patients who fulfilled the inclusion criteria and patients were recruited by their consultant in clinic or by post, so if patients did not attend their appointments or respond to postal invitation they could not be recruited.

The sample interviewed was the total recruited from the sample outlined above but, as alluded to in the discussion, this is an exploratory study which considers ethnicity and it is unlikely that saturation for analysis could be achieved without a larger sample and collection of detailed demographic data. Although the larger parent study sites collectively covered patient populations which included the South Asian population groups in UK, and the interview sample included participants from each of these groups, it was not possible to conduct useful comparisons within the broad South Asian and White groups without a larger sample, more detailed demographic data and therefore longer more in depth interviews.

Although interviews were conducted in the participant’s preferred language, by experienced bilingual interviewers where necessary, in order obtain quality data, this itself could be a source of bias through translation. Also, although many of the interviews and all the thematic analysis were conducted by an experienced researcher there could be the risk of bias due to misinterpretation.

This paper reports one of several elements to the larger diabetes and renal disease care pathway study which was a mixed method study addressing access and cultural competency on several levels. Whilst there are limitations to different elements of the research we suggest that the exploratory nature of the work together with the experience of the research team mitigate many of the risks outlined above for this qualitative element and that it is able to make useful observations and recommendations for future work.

## Conclusions and recommendations

The diabetic renal patients who have been referred for specialist renal care and found the referral process confusing have poor of awareness of kidney complications of diabetes.

Healthcare providers should be more aware of the ongoing information needs of long term diabetics as well as the context of any information exchange including language barriers.

More in depth research is required to understand the influence of ethnicity and culture on access to renal care.

## Competing interests

The authors do not have any competing interests.

## Authors’ contributions

EW carried out the data collection and analysis; GR led the design of the study and assisted with data analysis and interpretation; JF, KF, RG, and LL assisted with patient recruitment. All authors contributed to writing the paper. All authors read and approved the final manuscript.

## Pre-publication history

The pre-publication history for this paper can be accessed here:

http://www.biomedcentral.com/1471-2369/13/157/prepub
